# Brillouin–Raman micro-spectroscopy and machine learning techniques to classify osteoarthritic lesions in the human articular cartilage

**DOI:** 10.1038/s41598-023-28735-5

**Published:** 2023-01-30

**Authors:** Martina Alunni Cardinali, Marco Govoni, Matilde Tschon, Silvia Brogini, Leonardo Vivarelli, Assunta Morresi, Daniele Fioretto, Martina Rocchi, Cesare Stagni, Milena Fini, Dante Dallari

**Affiliations:** 1 Department of Chemistry, Biology and Biotechnology, University of Perugia, 06123 Perugia, Italy; 2 Reconstructive Orthopaedic Surgery and Innovative Techniques-Musculoskeletal Tissue Bank, IRCCS Istituto Ortopedico Rizzoli, Via G.C. Pupilli 1, 40136 Bologna, Italy; 3 Surgical Sciences and Technologies, IRCCS Istituto Ortopedico Rizzoli, Via Di Barbiano 1/10, 40136 Bologna, Italy; 4 Department of Physics and Geology, University of Perugia, Via A. Pascoli, 06123 Perugia, Italy; 5 CEMIN-Center of Excellence for Innovative Nanostructured Material, 06123 Perugia, Italy; 6 Scientific Director, IRCCS Istituto Ortopedico Rizzoli, Via Di Barbiano 1/10, 40136 Bologna, Italy

**Keywords:** Optical techniques, Osteoarthritis

## Abstract

In this study, Brillouin and Raman micro-Spectroscopy (BRamS) and Machine Learning were used to set-up a new diagnostic tool for Osteoarthritis (OA), potentially extendible to other musculoskeletal diseases. OA is a degenerative pathology, causing the onset of chronic pain due to cartilage disruption. Despite this, it is often diagnosed late and the radiological assessment during the routine examination may fail to recognize the threshold beyond which pharmacological treatment is no longer sufficient and prosthetic replacement is required. Here, femoral head resections of OA-affected patients were analyzed by BRamS, looking for distinctive mechanical and chemical markers of the progressive degeneration degree, and the result was compared to standard assignment via histological staining. The procedure was optimized for diagnostic prediction by using a machine learning algorithm and reducing the time required for measurements, paving the way for possible future in vivo characterization of the articular surface through endoscopic probes during arthroscopy.

## Introduction

Bone and cartilage are specialized connective tissues organized in complex architectures designed both to support our body and allow its locomotion, thanks to muscles insertions. The synergy between bone and cartilage tissues is crucial in the joint, where the hyaline cartilage covering the bone turns into a bearing-like structure, shaped in form of a thin deformable layer reducing compressive forces and simultaneously avoiding bones’ friction^1,2^. Under normal physiological conditions, the articular cartilage can perform these essential biomechanical functions with little damage or wear over the human lifespan. However, since it has a limited capacity for repair, lesions or injuries may lead to damage and therefore joint degeneration^3^.

Osteoarthritis (OA) is the most common form of peripheral joint arthritis affecting 7% of the global population, more than 500 million people worldwide^4^. Although it usually arises with advancing age, it is increasingly recognized that also younger people are impacted by OA, especially in the Western country, where overweight and obesity, often related to a sedentary lifestyle, and metabolic diseases, are considered key risk factors in the progression of cartilage degeneration^5^.

A variety of treatment options are available to patients with early OA, consisting of conservative management of the symptoms using both non-pharmacological (e.g., weight loss, lateral wedge insoles, bracing, and physical therapy), and pharmacological therapies (e.g., nonsteroidal anti-inflammatory drugs and intra-articular injections of active viscosupplement compounds or steroids). Unfortunately, this conservative care of symptomatic OA usually allows reaching modest or unsatisfactory results in medium/advanced cases and pain relief is shortly transient and more interventions are needed^6,7^. Therefore, an early diagnosis is a fundamental step to significantly increase the therapeutic efficacy and drastically slow down the progression of the pathology and the need to resort to prosthetic replacement.

During the orthopaedic check-up, the presence of OA is evaluated based on evident symptomatology (e.g., joint movement limitations, crepitus, joint effusions and tenderness), and is commonly confirmed by radiologic assessment. Changes on plain radiographs allow to highlight narrowing of the joint space, increased density of subchondral bone, or the presence of osteophytes. However, although this radiologic method allows the global analysis of the joint health state, it does not have a high resolution, and may present artefacts since the position assumed by the patient during the procedure can easily distort the distance between the bones in the joint site. Therefore, although the radiograph based diagnostic method is cheap and readily available, it is considered not effective enough in the detection of the OA early stages^8,9^. Computed tomography (CT) and magnetic resonance imaging (MRI) may be helpful in the evaluation of the early stages of degenerative joint disease. Nevertheless, generally MRI procedures have good validity for diagnosis of deep cartilage lesions, while they are relatively inaccurate for evaluation of low grade lesions or validation of intact cartilage surface^10,11^. Weight-bearing Cone Beam CT combines the high resolution of a CT with the possibility of analyzing joints in their daily use position: it is mainly used for knee and ankle^12,13^, and let to achieve information on the distance between subchondral bone surfaces. However, high costs, and the use of ionizing radiations, are some drawbacks of these techniques which have to be taken into account. Ultrasound (US) is a valuable and attractive tool for imaging information about articular cartilage, synovitis, periarticular soft tissues and bony cortical alterations in peripheral OA joints^14^. Nevertheless, the most relevant drawback of US in OA consists of its partial accessibility to the inner joint structures, resulting in frequent difficulties in the complete visualization of the hyaline cartilage in most joint sites. Moreover, the lack of standardized definitions and scoring systems for all findings is another important limitation to take into account^15^. Finally, also arthroscopy is used in selected patients as a technique to enable defining low-grade damages on the cartilage surface through visual inspection by the orthopedist. However, in a context of an early-stage OA, the cartilage lesions are still at a cellular level and structural damages are hardly detected^9,16^. In cases in which the arthroscopic procedure is considered the treatment of choice especially in young patients with an established articular, meniscal or ligament lesions, the evaluation of the damaged area with a probe coupled with additional tools able to thoroughly detect tissue degeneration signs might be useful to diagnose an early-stage of tissue degeneration and, consequently, to prevent the silent progression of the OA disease. To this aim, Brillouin and Raman micro-Spectroscopy (BRamS), a correlative technique not destructive, contact-less and not in need of labelling or contrast agents, based on the inelastic scattering of light from condensed matter^17^, might be used. Due to its capability to perform the chemo-mechanical characterization of the biological tissues, without interfering with them, it has the potential of being implemented in probes, supporting the orthopaedist in the formulation of the correct diagnosis during the arthroscopy. Specifically, in BRamS the detection of the macromolecular vibrations (via Raman scattering) is coupled with the simultaneous evaluation of the acoustic wave propagation (via Brillouin scattering) in the material, achieving samples’ chemo-mechanical phenotyping with micrometric spatial resolution and allowing a wide range of application in the biomedical field^18,19^. In recent years, this technique has been successfully used to examine different biological entities—i.e., single-cells, tissue-phantom models based on biopolymeric hydrogels, microbial biofilms and tissues—and several pathological conditions, such as the corneal keratoconus, the amyloid plaques in Alzheimer's disease and pre-cancerous lesions in Barrett’s oesophagus^20–25^. Furthermore, in our previous works, we used BRamS for the characterization of musculoskeletal healthy tissue sections (i) belonging to different histological types and anatomical regions, such as the articular cartilage and both the cortical and trabecular bone tissues in the diaphyseal and the epiphyseal regions of the human femur in a healthy status^26–28^ and (ii) treated with different sample processing, such as the freezing at − 80 °C and the paraformaldehyde fixation^29^.

Here, we present a proof-of-concept study that potentially constitutes an important step towards the application of the BRamS to the field of diagnostic and prognostic research on orthopaedic diseases. Thanks to the previously acquired knowledge in healthy tissue phenotyping, the effects of OA progression were revealed in some resections of femoral heads, excised from patients who underwent a total hip replacement. Specifically, measurements collected on both the articular cartilage surface and the subchondral bone were compared with the results obtained by histological assessment, unveiling the capability of this method to describe the tissue damages in agreement with these techniques. In particular, the Brillouin spectrum has proven to contain mechanical markers of the development of the disease, which were used (i) to compare the structure of areas of the articular cartilage and the subchondral bone subjected to major and minor loads inside the joint cavity and (ii) to characterize the articular surface of resections affected by different degree of OA degeneration (OA grading) and assigned by histological evaluation.

The possibility to recognize grades and extension of the damage in the portion of the articular surface exposed to the synovial cavity is a great advantage for in-vivo applications for an early OA diagnosis. In this respect, we can infer that adapting common arthroscopic probes with spectroscopic detection systems would mean performing a more accurate analysis than other mechanical-probing techniques (e.g., US), since BRamS can simultaneously assess cartilage mechanics and chemistry with a subcellular resolution^23^.

Furthermore, the traditional Brillouin spectral analysis was aided by multivariate statistical approaches—e.g., Principal Component Analysis (PCA)—to disclose the origin of the differences discovered in the mechanical make-up of sections with a progressive OA grading. Finally, a supervised machine learning (ML) algorithm based on Linear Discriminant Analysis (LDA) was built up to automatically classify the collected Brillouin spectra, according to the degree of severity of the pathology, thus reproducing a standardized procedure required for a novel diagnostic/prognostic tool, easily viable also by personnel not qualified in spectral data recognition. This method has shown a global accuracy in the single-spectrum prediction of better than 86%, which increases to even higher values in differentiating moderate and severe OA grades.

## Results

Five different biopsies—named here as biopsy#1 up to biopsy#5—were excised from the femoral head section of just as many patients undergoing unilateral total hip arthroplasty (UTHA). From each biopsy two different samples were selected from the anatomical portion subjected to the minor (LWB, infero-medial region) and major loadings (MWB, supero-lateral region), thus obtaining a total number of ten samples (two for each patient). More details are given in “Materials and methods” (“Patients and sample collection”). After spectroscopic analyses, each section underwent histological processing and evaluation to assess the degree of severity of the osteoarthritic lesion, as described in the “Materials and methods” (“Histological and histomorphometric analyses”).

### Chemo-mechanical characterization of the human femoral head in healthy conditions

**Figure 1:**
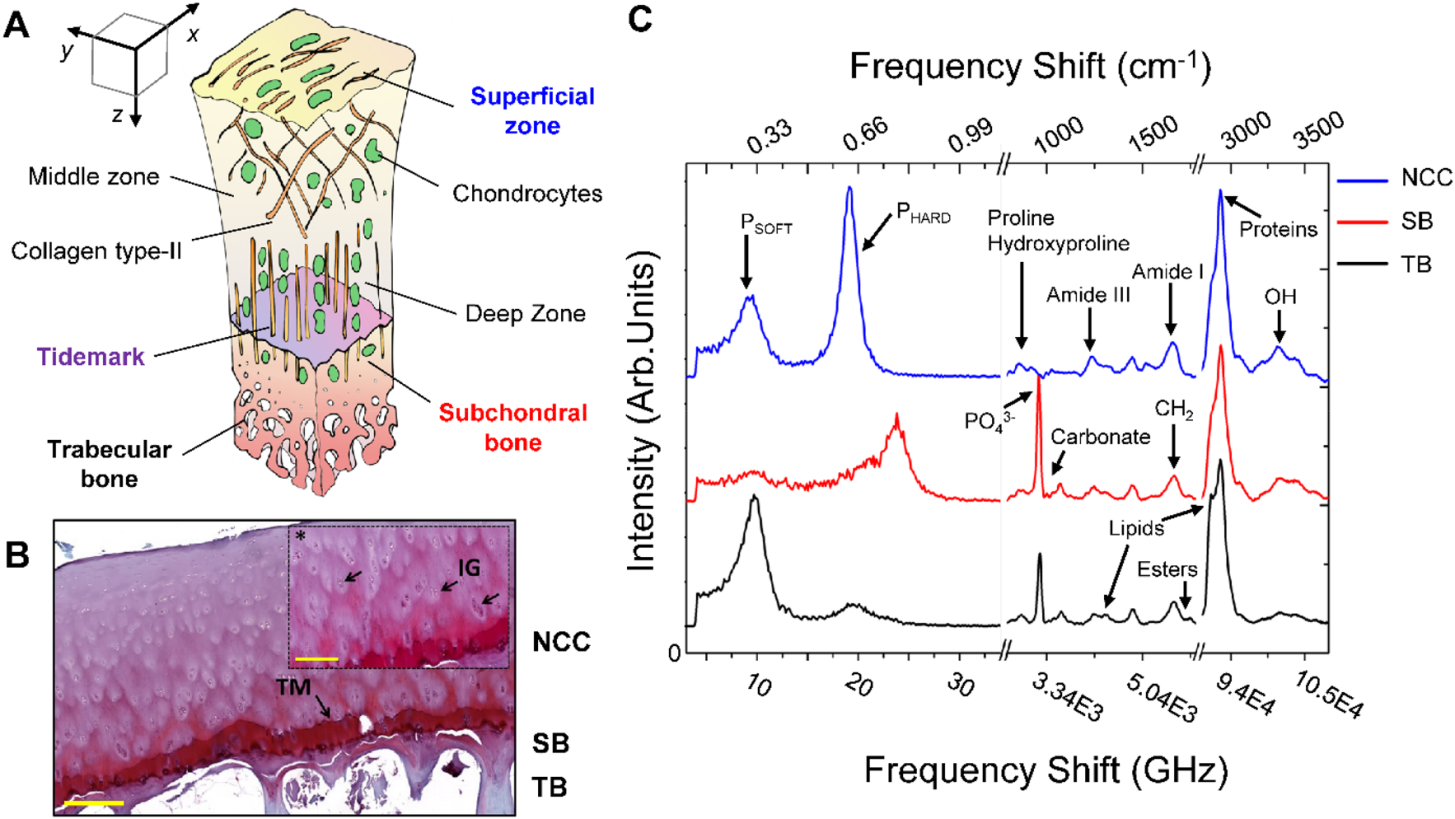
Chemo-mechanical and histological characterization of the human femoral head in a healthy condition. (**A**) Graphical sketch describing the most important biological structures constituting the femoral sections analysed in both the articular cartilage layer and the bone structure below. (**B**) Safranin-O/Fast Green staining of the least weight-bearing (LWB) of biopsy#1 (0-none Kallgren–Lawrence scale); scale bar 300 μm and 200 μm (*); *TM* tidemark, *NCC* non-calcified cartilage, *SB* subchondral bone, *TB* trabecular bone, *IG* isogen groups; (**C**) Typical Brillouin and Raman reconstructed spectra collected in the non-calcified cartilage surface (NCC-blue), in the subchondral bone (SB—red) and trabecular bone (TB-black) of the LWB section of biopsy#1 along with the signatures of characteristic Brillouin and Raman bands of interest.

A graphical sketch depicting the articular cartilage and subchondral bone structures with their principal biological constituents of a healthy femoral head is reported in Fig. 1A. Following the z-direction, from the superficial layer of the cartilage (xy plane) down to the trabecular bone inside the femoral epiphysis, different tissue organizations are found, characterized by proper chemical composition and distinctive cells-to-fibres relative ratios. The superficial zone of the cartilage in contact with the synovial joint is formed by flat chondrocytes squeezed between compact bundles of collagen; the middle zone is constituted by rounded chondrocytes, organized in the so-called isogen groups (IG) and submerged in a relatively disorganized mesh of collagen and proteoglycans; the deep-zone can be easily recognized for the presence of chondrocytes disposed in columns along the axis of the collagen fibres, which provides the anchorage of non-calcified cartilage (NCC) layers to the underlying mineralized ones. Here, the tidemark (TM) acts as a border between the articular cartilage and the mineralized tissues—i.e., the thin layer of calcified cartilage, the subchondral bone plate, presenting the typical lamellar distribution of cortical bone tissue type, and the trabecular bone forming the inner epiphyseal portion^30^. This layered organisation can be easily observed in the histologic assessment of the least weight-bearing section (LWB) of biopsy#1 in Fig. 1B, selected among the others as a healthy tissue reference (Mankin score, 5 ± 0.0) in agreement with the orthopaedic evaluation, which previously referred to it as a grade-zero (none) or one (doubtful) in the Kallgren–Lawrence (KL) scale—i.e., the first formalized attempts at establishing a radiographic classification scheme of OA^31^. Figure 1C reports the typical Brillouin (on the left of the break) and Raman (on the right) spectra collected in different points of the healthy femoral epiphysis before the histological analysis, namely the articular superficial layer (NCC-blue), the subchondral bone plate (SB-red) and the trabecular bone (TB-black), along with the assignations of Brillouin and Raman peaks of interests.

Brillouin peaks arise from the interaction of light with thermally activated acoustic waves propagating in the matter, thus providing its elastic characterization through the peaks’ intensity (proportional to the elastic species concentration) and frequency shift ν_B_ (proportional to the velocity of the acoustic wave in the elastic species)^18^. In particular, Fig. 1C evidences that the Brillouin spectra collected on both the cartilage and the bone tissues are characterized by at least two different peaks, namely P_SOFT_ and P_HARD_, ranging between 4 and 13 GHz and 13 and 34 GHz respectively, with different relative ratios and average frequency shift going from one region to the other, confirming our previous findings in Refs.^26–28^. Their simultaneous presence in the same spectrum unveils the coexistence of micro-heterogeneities with different elastic moduli in the same scattering volume (for further details see “Materials and methods”)^27,29,32^. In our previous works on the characterization of human healthy samples of bone and cartilage tissues, the detection of these two components was found to be dependent on the coexistence at the micrometric levels of an ordered phase of collagen bundles with different levels of mineralization and orientations (P_HARD_), and a disordered phase of extracellular matrix, with poorly organized fibres, non-collagenous proteins and water, responsible for the cells survival and signalling inside the tissue (P_SOFT_). Furthermore, the investigation of tissue organization in both the diaphysis and the epiphysis of femoral bones has revealed that also the bone marrow constituents—i.e., red cells and adipocytes—contribute significantly to both P_SOFT_ intensity and frequency shift^27,28^.

Raman peaks originate from the interaction of light with molecular vibrations of the macromolecules present in the sample^33^. Specifically, their frequency shift and intensity retain information about the nature of molecular vibration and its concentration inside the sample, respectively (further details are given in “Materials and methods”). In Fig. 1C the spectrum of the NCC cartilage shows typical signatures of collagen bundles—i.e., the amide III and amide I vibrations of the peptidic bond at 1250 cm^−1^ and 1660 cm^−1^, the proline and hydroxyproline ring vibrations at 853 cm^−1^ and 940 cm^−1^—while the subchondral bone plate (SB-red) and the trabecular bone (TB-black) spectra contain also signals from the inorganic phase of hydroxyapatite crystals present in the architecture of the mineralized collagen bundles—i.e., the phosphate group vibration at 965 cm^−1^ and the carbonate group substitutions at 1064 cm^−1^. Finally, the typical spectrum collected in the trabecular bone of the epiphysis unveils the characteristic signature of the lipidic component coming from the bone marrow constituents—i.e., the signal of CH_2_ twisting at about 1310 cm^−1^, the lipidic CH_2_ stretching at 2845 cm^−1^, and the C=O vibration of esters at 1780 cm^−1^^34–36^.

### Monitoring the OA development in the articular cartilage surface and subchondral bone of the least weight-bearing (LWB) and most weight-bearing (MWB) sections in a single-patient biopsy

**Figure 2:**
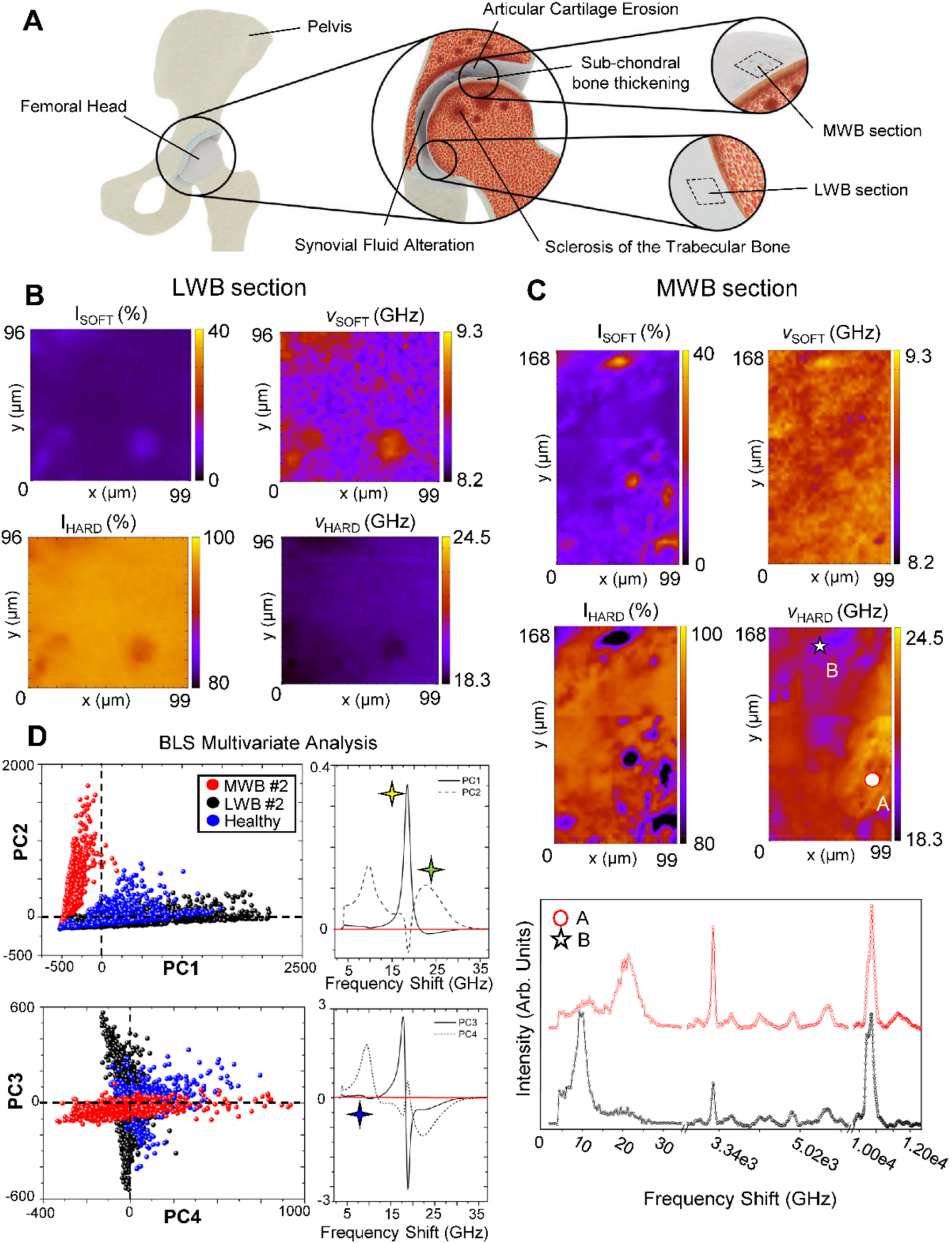
Mechanical imaging of the articular cartilage surface of the least weight-bearing (LWB) and most weight-bearing (MWB) sections of biopsy#2, revealing the main differences in their elastic properties. (**A**) Graphical sketch depicting the hip joint structure with a focus on the infero-medial (LWB) and supero-lateral (MWB) anatomical regions of the femoral head, along with the major manifestations of OA development on both the articular cartilage surface and the bone tissues. (**B**) Maps of the distributions of the relative percentage of P_SOFT_ and P_HARD_ components, namely I_SOFT_ and I_HARD_, with their respective frequency shift ν_SOFT_ and ν_HARD_ in the articular cartilage surface of the section less subjected to mechanical stresses (LWB section). (**C**) On the top, maps of the distributions of the relative percentage of P_SOFT_ and P_HARD_ components, namely I_SOFT_ and I_HARD_, with their respective frequency shift ν_SOFT_ and ν_HARD_ in the articular cartilage surface of the section subjected to major loads (MWB section). On the bottom, Brillouin and Raman single-point spectra (A-red circle and B-black star) collected in regions of interest on the map. (**D**) Brillouin spectral analysis by means of the principal component analysis (PCA), revealing the main differences among the spectra collected in the section subjected to the least weight-bearing (LWB #2—black), to the most weight-bearing (MWB #2—red) of sample#2, and the reference healthy phenotype (Healthy—blue).

OA development involves all components of the joints, from the articular cartilage and the subchondral bone to the synovial cavity, muscles and ligaments. The graphical sketch of Fig. 2A summarizes the primary manifestations occurring at the hip joint interface, on both the infero-medial (LWB) and supero-lateral (MWB) portion of the human femoral head, including the progressive erosion of the protective layer of articular cartilage due to biomechanical and inflammatory mechanisms, the thickening in the subchondral bone plate with an increase of bone remodelling processes, and sclerosis in the trabecular bone. It is worth noting that, the abnormal mechanical stresses acting on the surface of the cartilage during the patient lifespan constitute the primary triggers for the OA onset, generating physical micro-modifications, hence the chemo-mechanical examination of the cartilage superficial layer potentially retains markers of the disease’s early manifestations^1,2^.

Figure 2B,C show two Brillouin maps collected respectively on the articular surface of the section anatomically subjected to a minor (least weight-bearing, LWB) and major loadings (most weight-bearing, MWB) of the same patient (biopsy#2, for further information, see “Materials and methods”). Specifically, the distributions of the relative volume occupied for both the P_SOFT_ and P_HARD_ components (I_SOFT_ and I_HARD_) are shown together with the distribution of their respective frequency shift values (ν_SOFT_ and ν_HARD_). The relative percentage intensities were calculated considering both the coexistence of the soft and hard peaks in the same volume and a filling factor, which takes into consideration the effect of signals intensity modulations due to the tissue's porous surface. Moreover, the two components were weighted considering the different scattering efficiencies of the elastic species involved following the procedure reported in Ref.^27^ and the “Materials and methods” (“Brillouin data analysis” section). It is worth noting, that this biopsy was chosen for imaging because it showed among the other evident morphological OA manifestations at the macroscopic inspection (further details are provided in Supplementary Fig. S1 of Supplementary Materials).

The LWB section (Fig. 2B) is characterized by Brillouin spectra with very low contributions from the I_SOFT_ component—i.e., the cellular phase—localized in a few circularly-delimited zones presenting a frequency shift ν_SOFT_ around 8.7 GHz. Conversely, the contribution from the fibrillar component I_HARD_ is predominant, filling the spaces among the softer regions. The frequency shift ν_HARD_ is rather homogeneous and centred around 18.5 GHz, thus revealing values similar to those reported in Fig. 1C for the articular cartilage spectrum of healthy tissue (NCC-blue spectrum). The section more subjected to load MWB (Fig. 2C) is characterized by higher contributions from the I_SOFT_ component, which is, however, distributed in irregular shapes and characterized by an almost homogeneous shift of ν_SOFT_ to higher values (around 9.1 GHz). This could suggest a loss of integrity and an overall alteration in both the cellular morphology and mechanical properties, likely due to the depletion of the cartilage superficial layer. Furthermore, in the MWB section (Fig. 2C) the contributions of the hard intensity I_HARD_—i.e., the ordered phase of fibres—filling the regions between the softer areas spans a wide range of frequency shifts values (ν_HARD_), ranging between 19 and 24 GHz, resembling values similar to the ones shown in Fig. 1C) by the subchondral and the trabecular bone spectra (SB-red and TB-black spectra), and thus suggesting a complete depletion in the articular cartilage layer covering the mineralized tissues. This is confirmed by the Brillouin and Raman single-point spectral analysis of regions A (red circle) and B (black star), selected as extreme values of the ν_HARD_ range, which shows in both the cases, typical signals of hydroxyapatite (v_1_ PO_4_^3−^ at 965 cm^−1^) and lipids (1301 cm^−1^), resembling spectral features similar to the cortical bone (Fig. 1C-SB) and the trabecular bone (Fig. 1C-TB) tissue types, respectively. It should be noted that the exposition of mineralized tissues, both the calcified cartilage layer and the subchondral bone underneath, denotes an advanced disease state in the MWB section, compatible with a grade 4 (severe) in the Kellgren-Lawrence classification of OA^31^.

Finally, Fig. 2D reports the result of the Principal Component Analysis (PCA) applied to a dataset composed of thousands of Brillouin spectra collected from the articular surface of LWB (black) and MWB (red) sections of biopsy#2 and from the articular surface of the reference for the healthy phenotype (blue), i.e., the LWB section of biopsy#1. PCA analysis is a multivariate statistical treatment used to reduce the dimensionality in a data set, through the extrapolation of principal components (PCs). This type of data mining technique has the notable advantage of providing a global view of the spectral features in the dataset when dealing with many measurements. Specifically, it allows detecting clusters of objects (spectra) grouped according to criteria of similarity in the individual variables (peaks) that describe them. Moreover, PCA, among the other techniques of multivariate statistics, provides discriminants in the distribution—i.e., the principal components (PCs)—more easily interpretable, allowing the qualitative evaluation of the spectral differences generating these clusters. It is widely used in Raman spectroscopy^37,38^, but up to the present, only rarely applied to the Brillouin spectrum (for further details see Materials and method section)^39^. The scatter plot at the top of Fig. 2D reporting the PC1 and PC2 scores—i.e., the most important components in terms of statistical variance—shows that the LWB section of biopsy#2 (black points) and the healthy phenotype (blue points) share similar features that clearly distinguish them from the MWB section (red points) of biopsy#2, suggesting that the region minor involved in the mechanical stress is also the less affected by the pathology. Furthermore, the loadings plots of both PC1 and PC2 components on the top-right of Fig. 2D precise the origin of this clustering, revealing that the LWB section and the healthy reference are characterized by the presence of signals from the non-mineralized collagen fibres (yellow star), while the MWB section of biopsy#2 presents peaks coming from both the cellular soft phase and the mineralized collagen bundles (green star). However, the scatter plot of PC3 and PC4 components at the bottom of Fig. 2D evidences that minor differences can be appreciated also between some spectra of the LWB (black points) section of biopsy#2 and the healthy section (blue points). In fact, the LWB phenotype is characterized by (i) spectra with greater variability in the frequency shift of the not-mineralized collagen peaks, viewable in form of a wider clustering of the black points along the PC3 axis and (ii) spectra without the signal from the Brillouin soft component, verifiable in the PC4 loadings plot (blue star). This analysis underlines that even if the region subjected to minor load (LWB) of biopsy#2, discloses mechanical characteristics similar to the healthy section, it preserves some peculiar spectral features—i.e., a greater contribution from the fibrous component of the tissue—that suggest manifestations of the onset of cartilage degeneration.

**Figure 3:**
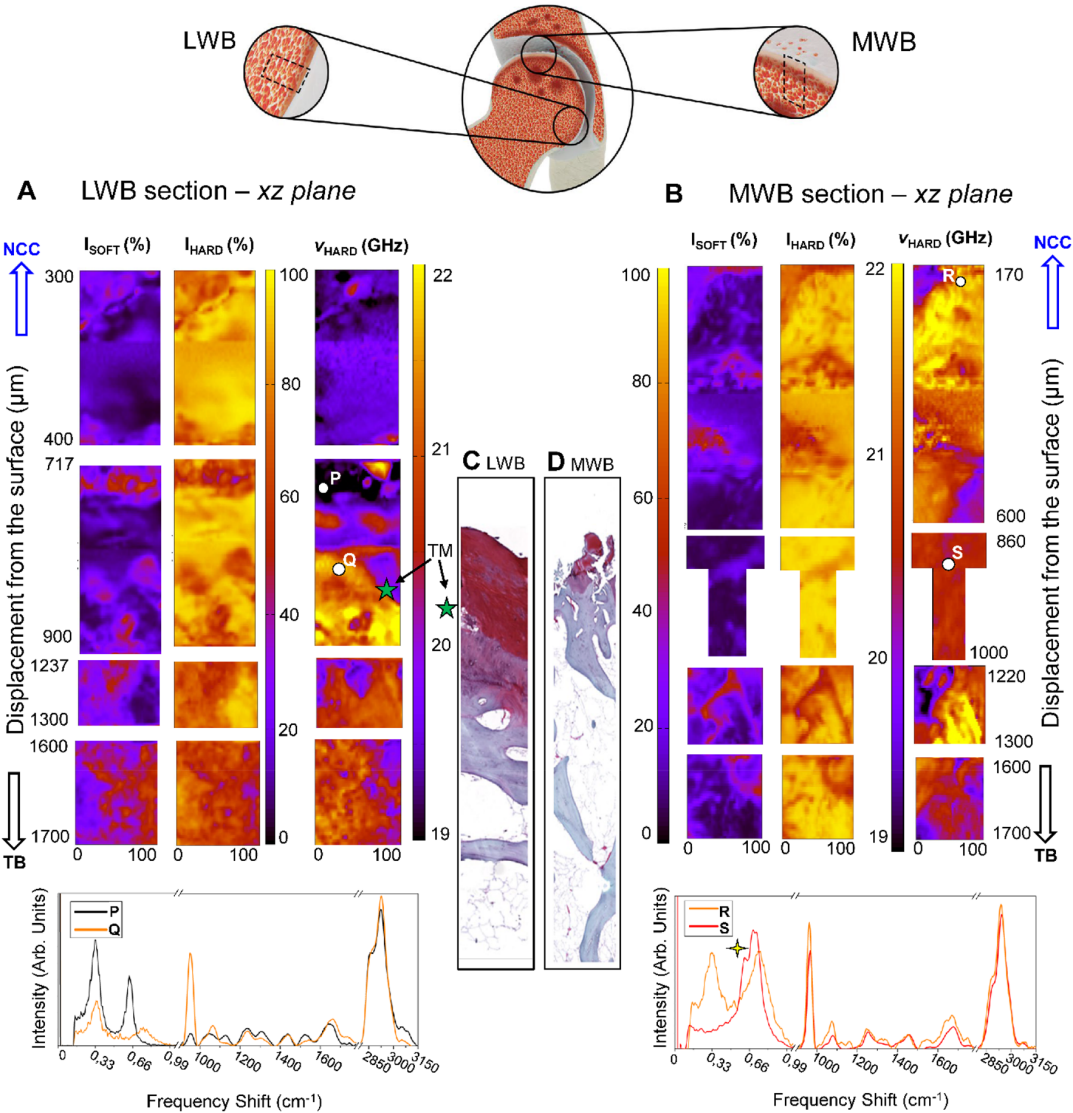
Mechanical imaging of the least weight-bearing (LWB) and most weight-bearing (MWB) sections in the xz plane of the non-calcified cartilage (NCC) and the subchondral and trabecular bone (TB) of biopsy#2, revealing the main differences in their elastic properties. (**A**) At the top, Brillouin maps reporting the distribution of the relative percentage of P_SOFT_ and P_HARD_ components, namely I_SOFT_ and I_HARD_, with the frequency shift of the hard component ν_HARD_, collected in the LWB section of biopsy#2. At the bottom are Brillouin and Raman spectra of points of interest in the map (P-black, Q-orange). (**B**), At the top, Brillouin maps reporting the distribution of the relative percentage of P_SOFT_ and P_HARD_ components, namely I_SOFT_ and I_HARD_, with the frequency shift of the hard component ν_HARD_, collected in the MWB section of biopsy #2. At the bottom are Brillouin and Raman single-point spectra of points of interest in the maps (R-orange, S-red). The yellow star stresses the presence of a double-peaked signal in the BLS spectrum. Histological assessment of OA grade in the LWB (**C**) and the MWB sections (**D**) of biopsy#2 through Safranin-O/Fast Green staining. Green stars point out the TM-tidemark position.

To inspect the grade of OA development in underlying tissues of LWB and MWB regions of biopsy#2 and confirm the previous observations on the articular cartilage surface, the BRamS spectroscopic imaging was conducted at different depths going from the not-calcified layer of the cartilage (NCC) to the inner portion (TB) of the epiphysis (*xz* plane of Fig. 1A) along with the histological assessment through Safranin-O/Fast Green staining. The histological evaluation of OA tissues is usually done on the entire cartilage layer and the first part of the subchondral bone, to estimate the residual thickness of the cartilage cushion and its overall status. Figure 3 reports the Brillouin maps of the distribution of the P_SOFT_ and P_HARD_ components (I_SOFT_ and I_HARD_), along with the ν_HARD_ values, for both the LWB (Fig. 3A) and the MWB (Fig. 3B) sections of biopsy#2, respectively, and the histological analyses performed on the same side of the sections previously analysed through spectroscopic imaging in Fig. 3C,D.

Similarly to its articular surface, the section subjected to minor loads (LWB, Fig. 3A) of biopsy#2 is characterized by Brillouin spectra with a prevalence of the hard phase of fibres I_HARD_. The distribution of its frequency shift (ν_HARD_) ranges from 19 to 20 GHz, ascribable to the presence of a dense network of non-mineralized collagen fibres, and abruptly breaks around 800 μm to form a well-defined tidemark between non-calcified and calcified tissues (TM-green star). Moreover, smaller contributions from the softer component in the cartilage I_SOFT_, likely due to the cellular component and not-ordered extracellular matrix, are visible in a restricted area at about 300 μm from the surface and in the proximity of the deep zone, closely to the tidemark. Below this boundary, an increase in the soft component of the tissue is well visible, along with the parallel shift in the hard peak frequency (ν_HARD_) to higher values, consistent with the detection of signals from the subchondral cortical plate (SB) and trabecular bone (TB). The Brillouin and Raman single-point spectra in the bottom of Fig. 3A, collected above (P-black) and below (Q-orange) the tidemark confirms the sharp transition between the two regions, marked by the abrupt increase in the intensity of the ratio between the phosphate vibration ν_1_PO_4_^3−^ at 965 cm^−1^ and the CH_2_ wagging intensities (i.e., the collagen fibres’ mineralization degree)^35^. Despite a similar relative ratio between the soft (I_SOFT_) and fibrous (I_HARD_) content, the Brillouin map of the section subjected to major loads (MWB) in Fig. 3B shows sites of ossification in the articular cartilage layer already from 200 to 600 μm from the surface, clearly distinguishable by a shift of ν_HARD_ up to 22 GHz, so that residual fragments of the not-mineralized matrix may be found just in a small portion on the upper left area around 200 μm deep. This result is confirmed by the Brillouin and Raman single-point spectrum reported at the bottom of Fig. 3B collected in point R (orange), showing the typical Brillouin spectrum of cortical bone tissue (Fig. 1C-SB) and an intense Raman vibration coming from the hydroxyapatite crystals at 965 cm^−1^. Therefore, the lack of a precise and clear tidemark dividing non-calcified tissues from calcified tissues is evident. In addition to that, the regions located beyond 800 μm (coincident with the tidemark of the LWB section) are characterized by Brillouin spectra with a peculiar band shape, displaying simultaneously both signals from mineralized and non-mineralized fibres, as shown in the representative Brillouin and Raman single-point spectrum S (red) reported at the bottom of Fig. 3B, where a double-peaked band in the P_HARD_ region can be identified (yellow star). These micro-heterogeneities could be due to the presence in the scattering volume of a substantial part of collagen fibres recently deposited or undergoing bone resorption, due to the phenomena of increased tissue remodelling caused by OA. In this regard, it should be noted that this effect outcomes in a decrease in the averaged degree of mineralization of the subchondral region, as revealed by the lower ratio between the ν_1_ PO_4_^3-^ vibration and the CH_2_ wagging of spectrum S (red) with respect to spectrum R (orange), potentially denoting a decreased ability to resist mechanical damage of the bony tissues more affected by the OA progression.

**Figure 4:**
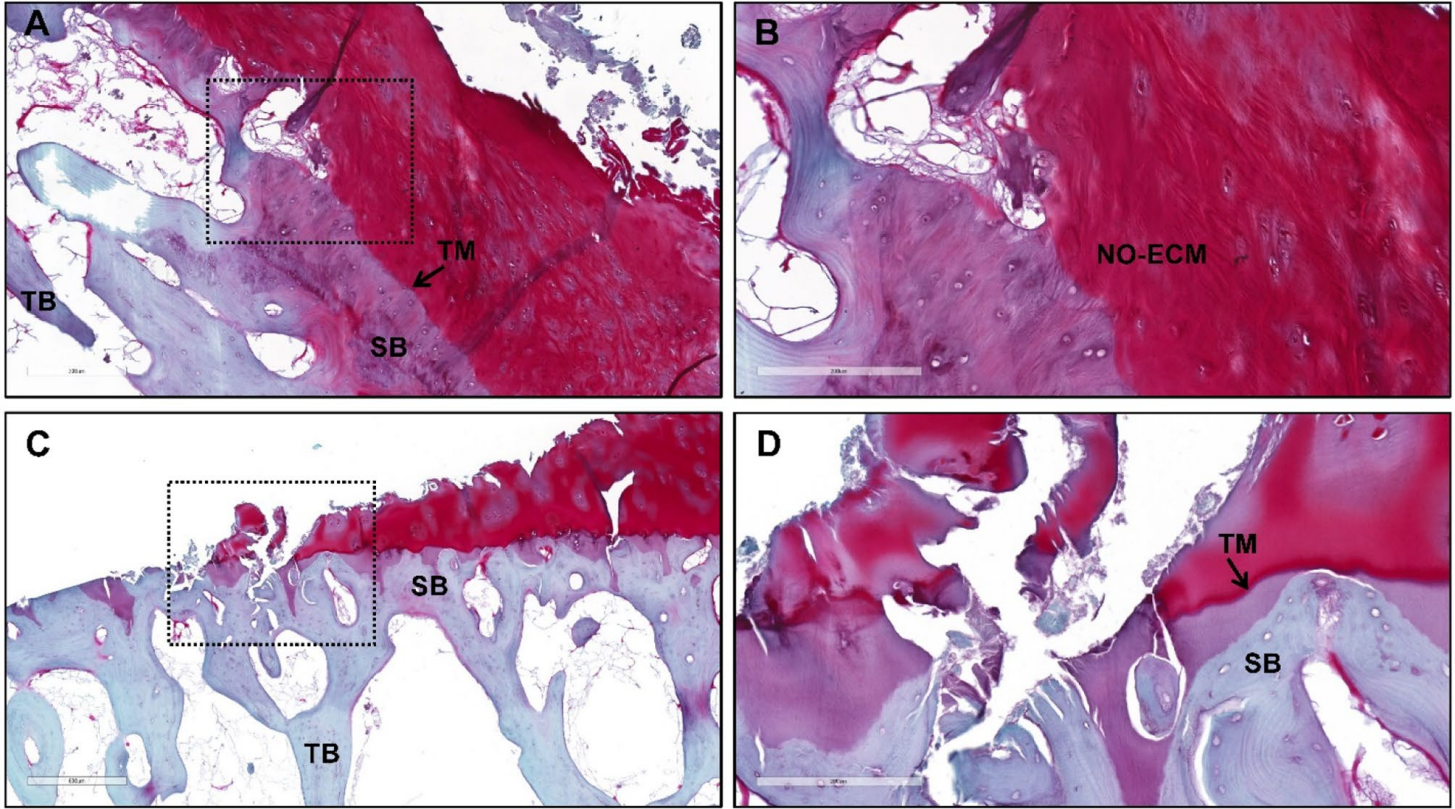
Histological images of the least weight-bearing (LWB; **A**,**B**) and most weight-bearing (MWB; **C**,**D**) of sample#2. *SB* Subchondral bone, *TM* Tidemark, *NO-ECM* non-ordered extracellular matrix, *Staining* Safranin-O/Fast Green, Magnification: ×8 (**A**, **C**), scale bar 300 μm; ×20 (**B**,**D**), scale bar 200 μm.

Safranin-O/Fast Green staining of LWB and MLB sections shown in Fig. 3C,D and more in detail in Fig. 4 below confirms our findings. Specifically, it evidences a well recognizable transition between the non-calcified articular cartilage and the subchondral bone (SB) marked by the tidemark (TM) as regards the LWB sample (Figs. 3C, 4A,B). Concerning the cartilaginous compartment, some irregularities due to the presence of discontinuities, fibrillation zones and mild erosion phenomena can be noted, as well as the presence of a non-ordered extracellular matrix lacking in the cellular component, confirming the suspected onset of cartilage degeneration (Mankin score 8.0). Otherwise, an extended and severe cartilage erosion characterizes the MWB sample (Figs. 3D, 4C,D ) getting to involve the mineralized cartilage and, in some zones to the level of subchondral bone, thus interrupting the TM continuity. Therefore, a defined border between non-calcified articular cartilage (NCC) and calcified cartilage is not present, with subsequent protrusion of SB toward the cartilaginous compartment. Furthermore, severe hypocellularity of the cartilaginous compartment is observed as well as loss of pericellular staining, which is an index of chondrocytes’ suffering and lack of matrix proteoglycans deposition (Mankin score 8.5).

The results obtained using Brillouin and Raman analysis sustains the capability of the technique to provide important information about bone and cartilage architecture, with a good morphological correspondence with the histological staining using Safranin-O/Fast Green. Furthermore, the regions subjected to major load (MWB) in the femoral head were found to present altered mechanical characteristics compared to the less stressed regions (LWB) in biopsy#2 (as well as in biopsies #1, #3 and #4, see Supplementary Fig. S2 in Supplementary Materials). This fact emphasizes the close correlation between joint stress and disease onset, confirming that the regions of the femoral head, less involved in the loading’s sustenance show commonly a lower degree of OA progression, reasonably due to a minor exposition to the mechanical friction with the pelvic acetabulum. They also underline the necessity, during the arthroscopic screening, to assess the severity of the lesion preferably in the supero-medial region of the patient’s femoral head.

### Classification of the OA grading of most weight-bearing (MWB) sections using multivariate statistical treatment of Brillouin spectra and the histological assessment

**Figure 5:**
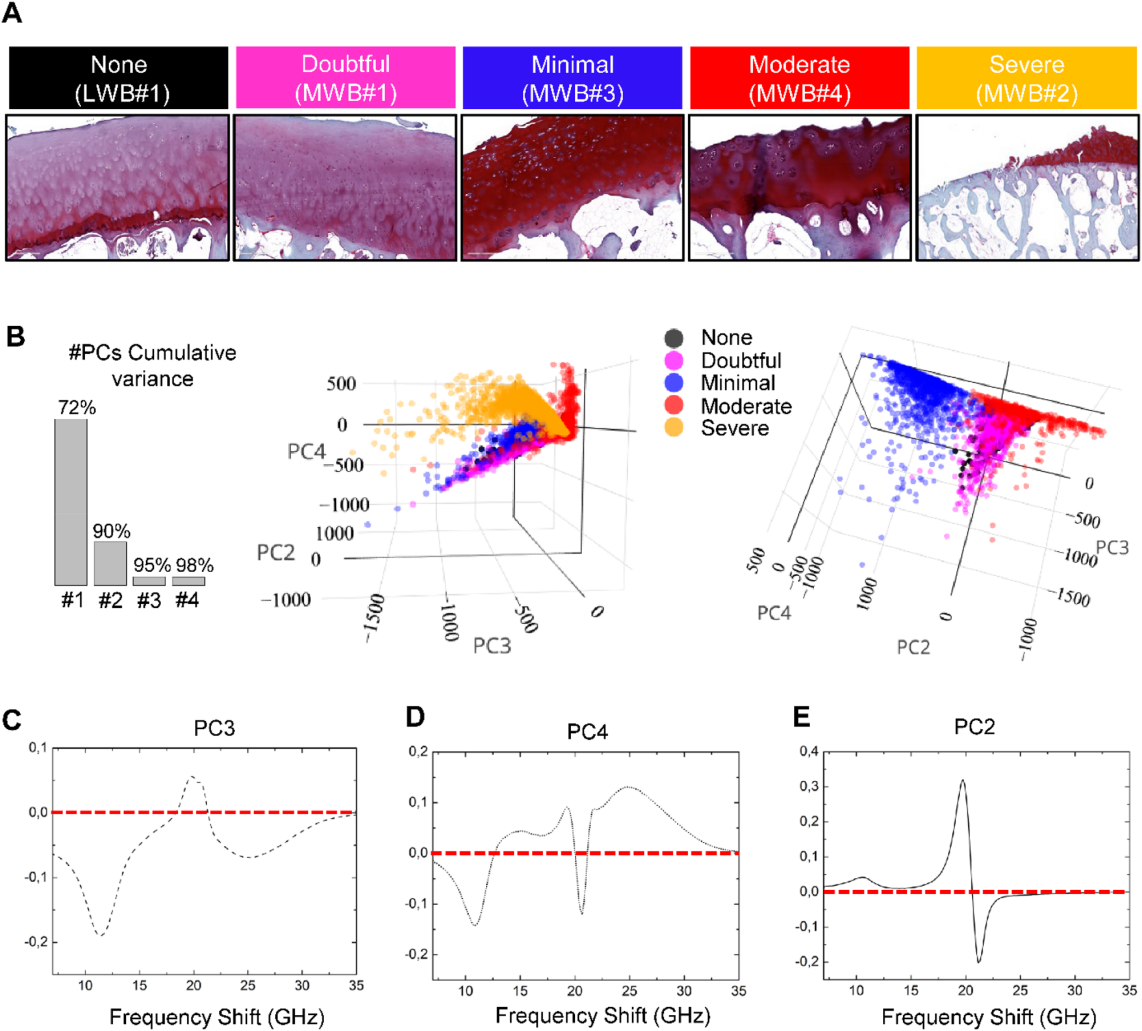
Principal component analysis of Brillouin spectra collected on the articular cartilage surfaces of MWB sections with different degrees of OA development. (**A**) Histological evaluation of OA progression in the articular surface of MWB sections of biopsy #1 to #4 ordered according to their increasing degeneration along with the healthy phenotype (LWB section of biopsy#1). (**B**) PCA scores plots of Brillouin spectra collected in the articular cartilage surface of the five sections, correspondent to the healthy phenotype (black) and four different grades of pathology severity, namely doubtful (magenta), minimal (blue), moderate (red) and severe (orange), along with the histogram reporting the values of PCs cumulative variance explained. PCA loadings plots of (**C**) the third (PC3), (**D**) the fourth (PC4) and (**E**) the second (PC2) components.

Once established that the assessment of the OA grade through histological staining on the xz plane’s femoral sections agrees with the results of the spectroscopic imaging of both the articular cartilage surface and its in-depth structure, the MWB sections (i.e. supero-lateral femoral head) of biopsy#1 to #4 were selected as representative of OA-affected tissues and their histologies ordered in Fig. 5A from the lightest grade (named doubtful, magenta) to the most severe grade (named severe, orange). The LWB section of biopsy#1—i.e., the reference used for the healthy phenotype (none, black) is reported for comparison. OA progression is characterized by the typical involvement of the cartilaginous compartment through superficial irregularities and/or superficial fibrillation. This may be accompanied by cell death or proliferation, an increase, or a decrease in matrix staining whereas the mid and the deep zone result are mostly unaffected, even if disorientation of chondron columns and cloning can occur (Fig. 5A—doubtful and minimal grades). As the OA becomes more extensive, delamination of the superficial layer and more serious erosion phenomena can occur involving the mid zone. Cyst formation or loss of fibrillary matrix in perichondral areas defined as “lacunar resorption” can be seen (Fig. 5A—moderate grade). Finally, severe OA is recognized by denudation, complete erosion of hyaline cartilage to the level of mineralized cartilage and/or bone. Typically, the bone at the denudated surface appears thicker and usually less mineralized than deeper trabecular bone and its surface can be accompanied by fibrocartilaginous repair (Fig. 5A—severe grade).

Brillouin spectra were detected from random points in the articular cartilage surface of the five different samples of Fig. 5A, collected in a dataset and analysed by PCA to reveal the major differences existing in their mechanical features. In fact, since the articular surface constitutes the first front in which the pathology develops, its structural state is the main parameter to assess the OA changes during arthroscopy. The results of the PCA analysis are reported in Fig. 5B–E where the objects are coloured according to the section of origin. It is worth noting that PC1 is not reported, since it reveals differences in the spectra mainly due to peaks absolute intensities, that can vary from one point to the other due to focalization differences on the samples’ rough surface and for this reason not useful for grade classification. The score plot on the left of Fig. 5B reveals that the spectra belonging to the severe grade (orange scores) are distinguishable from all the other ones, due mainly to both the third principal component (PC3) and the fourth principal component (PC4). The loadings plots of these two PCs respectively reported in Fig. 5C,D, highlight that the origin of this clustering is likely due to the presence of a shift to higher frequency values in the P_HARD_ of the Brillouin spectrum—i.e., the mineralized collagen bundles and the presence of an intense signal from the P_SOFT_ component—i.e., the not-ordered phase of extracellular matrix. This fact is not surprising, since the exposition of the mineralized tissues of the subchondral bone is a peculiarity of the highest grade of OA progression, while the other phenotypes (from 1 to 3) still maintain the presence of a cartilage layer, as clearly shown in the histologic assessment in Fig. 5A.

In the score plot on the right of Fig. 5B the orange points are masked, helping the reader to focus the attention on the other clusters, and disclosing that all the groups are well-differentiated thanks to the second (PC2) and the fourth (PC4) principal components, except for almost superimposed healthy (black) and the doubtful grade (magenta). More in detail, PC2 reported in the loadings plot in Fig. 5E distinguishes the doubtful (magenta) grade from the minimal one (blue), based on i) the detection of a shift to lower values of frequency in the not-mineralized collagen signal, and ii) the slight increase of the soft peak intensity in the minimal phenotype. These facts confirm an initial phase of remodelling and flaking of the extracellular matrix of the articular cartilage affected by the pathology, with a simultaneous rearrangement of the softer cellular component in the superficial layer. Conversely, PC4 divides the spectra with doubtful (magenta) and minimal (blue) grade phenotypes from the moderate one (red). The respective loadings plot in 5D) discovers the origin of the clustering in (i) an increase in the contribution from the signal from the mineralized collagen bundles and (ii) in a further reduction in the intensity of the soft component peak, when the OA phenotype degrades to moderate. These results suggest that the degeneration of the cellular component and the cartilage breakdown started in the doubtful and minimal phenotypes become higher, and simultaneously some local processes of ossification or exposure of the underlying bone have been triggered.

### LDA-based model for machine learning application to automatically discriminate OA grading through Brillouin spectra collected on the articular cartilage surface

The differences disclosed in the mechanical make-up of the tissue sections, affected by progressive grades of OA development, can constitute the basis of a machine learning (ML) algorithm, able to recognize and classify new spectra for diagnostic purposes. Machine learning techniques can be useful in the medical application because once the dataset has been catalogued and the model elaborated, they facilitate the automatic discrimination of new data even by non-technical personnel. Here, the same dataset reported in the previous paragraph and used for the PCA analysis was employed to train an ML algorithm based on Linear Discriminant Analysis (LDA), considering the five different categories previously pointed out, namely healthy (black), doubtful (magenta), minimal (blue), moderate (red), severe (orange). In detail, the database of spectra was split into two subgroups: a train-set containing 75% of the original data, randomly selected considering an equal percentage of each sub-groups, and a test-set collecting the remaining 25% of spectra. LDA analysis is a method for the reduction of the dimensionality of a dataset similar to a PCA. However, it is a supervised method that in addition to finding the component axes that maximize the variance of the data, also found the axes that maximize the separation between the labelled categories, usually allowing better performances of ML in the discrimination of the subgroups (more details are provided in the Materials and Method section)^39^.

**Figure 6:**
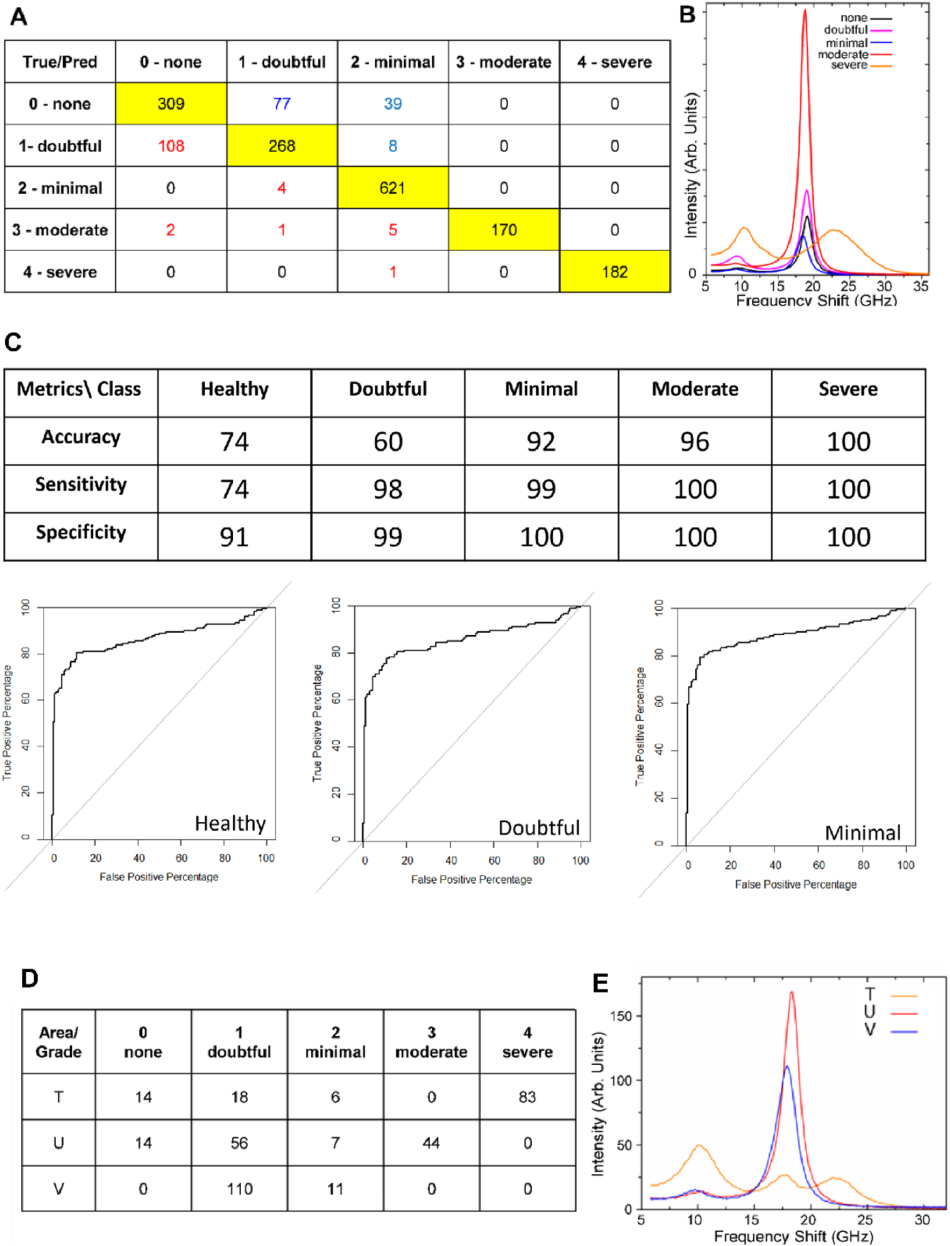
LDA modelling for machine learning application applied on the Brillouin spectra collected on the articular cartilage surface of MWB sections and LDA-based ML classification of fast Brillouin spectra collected in different areas of the articular cartilage (MWB-sample#5). (**A**) Confusion matrix obtained by the application of the LDA-based ML algorithm to the test-set correspondent to the remanent 25% of spectra selected. The right assignments are given in the yellow boxes along the diagonal, where false-negative and false-positive are red and blue coloured, respectively. (**B**) Averaged spectra of each category, namely, none (black), doubtful (magenta), minimal (blue), moderate (red) and severe (orange) used in the model. (**C**) Table reporting the main model metrics calculated on the five classes (top) along with the ROC curves for the healthy, doubtful and minimal phenotypes, respectively. (**D**) Results of the classification of fast spectra collected in different areas of the MWB section of sample#5, namely T (orange), U (red) and V (blue), along with the (**E**) averaged spectra of each group.

The ML-LDA based model elaborated on the train-set was then used to predict the class of the spectra contained in the test-set. The results of the predictions are reported in the confusion matrix of Fig. 6A along with the averaged spectra of the measurements in each category in the model (Fig. 6B). The matrix reports the predicted assignment of each spectrum in the rows and its true class in the column, resulting in that the principal diagonal (yellow boxes) reports the number of spectra for each class correctly assigned, whereas the false-negative and the false-positive outcomes are reported in the red and blue characters respectively.

The confusion matrix demonstrates that this simple model based on the spectral feature of Brillouin peaks collected on the surface of the supero-medial cartilage, reaches a global accuracy in the single-measurement prediction of about 86%, providing potential precious information for the orthopedist during the diagnostic procedure. Furthermore, it is interesting to note that most cases of misclassification involve the healthy and doubtful category, while a negligible number of spectra belonging to the minimal, moderate and severe phenotypes have been underestimated in severity. Accordingly, Fig. 6C reports that the global accuracy of 86% in the classification algorithm reaches considerably higher values for the minimal (92%), moderate (96%) and severe (100%) categories. In addition to that, the sensitivity (the capability to not underestimate the pathology grade) was found to be even higher for the three above mentioned categories and for the doubtful class (98%); likewise, the specificity (the capability to not over-estimate the pathology grade) was higher than the 90% for all the categories. Finally, these results are reinforced by the ROC curves shown for the healthy, doubtfull and minimal classes (not reported for the moderate and the severe categories since in those cases the accuracy is near to the 100%): the model performance in the classification issue—i.e., the area under the curve, progressively increases with the worsening of the grade pathology. Despite the high accuracy obtained by the algorithm, an important parameter to be optimized in the diagnostic procedure will be the time required for the single measurement. The reduction in the acquisition time of the spectrum, necessary to sample a wide area of the cartilage layer during the arthroscopic procedure, also abates the spectral signal-to-noise ratio, possibly affecting the accuracy of the algorithm. To test the validity of the LDA-based ML algorithm also with reduced time of acquisition, several spectra were acquired for 10 s in the articular surface of the MWB section of biopsy#5 (see “Materials and methods”). In detail, a series of measurements were collected on three different areas of the section distant centimetres one from the other and named here as T, U and V, simulating a diagnostic examination. Then each spectrum was classified by the ML algorithm into the predicted class, revealing the heterogeneous scenario reported in Fig. 6D, and unveiling the capability of the algorithm to recognize the Brillouin spectral shape also in case of fast measurements. The T portion of the MWB section presents numerous spectra recognized as severe progression, thus indicating that this area is characterized by the exposition of the subchondral bone layer. This result is stressed in Fig. 6E by the averaged spectrum of the T area (orange), reporting a clear signal from the P_HARD_ component of mineralized collagen bundles. Conversely, according to the algorithm, the V portion presents most spectra belonging to the doubtful phenotype and few spectra to the minimal one, thus disclosing an area of the sample, which is potentially less affected by the pathology, as also confirmed by its averaged spectrum (Fig. 6E, blue), showing peaks from both the cellular phase and the not-mineralized collagen. Finally, the U portion presents a mechanical make-up that is intermediate between the ones detected in the other two areas, since a comparable number of spectra presenting the minimal and the moderate phenotypes was found. This evidence suggests that the U portion is an extremely heterogeneous region, that is likely going towards osteoarthritis degeneration to moderate grade, characterized by a loss in the cartilage cellular component (Fig. 6E, red). This multiple grade pattern in the pathology progression in a wide area of the articular surface was observed in other femoral resections (Fig. S3) and analyzed more deeply in the Supplementary Materials.

## Discussion

In this proof-of-concept study, we show that Brillouin and Raman micro-Spectroscopy can reveal the main manifestations of hip OA progression in the human femoral head, with a good correspondence with the grade assessment through a more traditional technique—i.e. the radiological KL scoring and histological staining. Specifically, the mapping of both the articular surface and thickness of biopsies, procured from patients who underwent a unilateral total hip replacement, was found able to detect markers of articular cartilage degradation and ossification processes due to the OA onset. Moreover, the mechanical characterization of the articular cartilage surface, obtained by the combination of the solely Brillouin spectroscopy and multivariate statistical approaches—e.g., PCA—discloses that the anatomical portion of the femoral head less involved in loads (LWB) sustenance is commonly less severely affected by the development of the disease with respect to the ones more subjected to the compressive forces (MWB), confirming the strict relationship between mechanical insults and OA development.

Furthermore, the PCA analysis, performed on Brillouin spectra collected on the articular cartilage of sections presenting a progressive higher grade of OA degeneration, previously assessed by histological staining, demonstrates the capability to distinguish four different grades of pathology severity, namely doubtful, minimal, moderate and severe, based on modulation of both the relative intensities and frequency shifts of the soft (i.e., cellular phase and the not-ordered phase of the extracellular matrix) and hard (i.e., fibres content) components of the tissue. Specifically, going from the doubtful to the moderate grades was recorded (i) an initial step of increase (minimal), followed by a decrease (moderate) in the contribution of the P_SOFT_ peak, likely due to remodelling in the cellular and the not-ordered phase of the extracellular matrix organization, and (ii) a softening in the elastic moduli of the collagen fibres, probably due to a loss of integrity or to a more disordered fibrillar distribution. Finally, the severe grade is characterized by the detection of signals from the mineralized collagen bundles, due to the exposition of the subchondral bone and/or the ossification of the not-mineralized layers of the cartilage.

The application of the technique for the OA-grade assessment, using just the articular surface of the supero-lateral cartilage is a notable advantage because this anatomical area could potentially be reached in vivo using arthroscopic probes, supporting the common visual inspection by the orthopedist and excluding the need for more invasive biopsies. Although there are mechanical techniques currently being developed for probes—i.e., ultrasound-based technique^9^—Brillouin spectroscopy allows obtaining a spatial resolution in the order of the single cell. Furthermore, since the Brillouin spectrum alone was found sufficient to classify the pathology expression, the analysis of the Raman spectrum sometimes compromised by fluorescence phenomena, could be additionally performed but is not strictly necessary. The analysis of different portions of the supero-medial articular cartilage surface has also emphasized the possibility to reveal the extension of the lesion (i.e., staging) and help to define the overall impairment of articular cartilage. The evaluation of the mechanical phenotype in distinct areas can be employed by the surgeon to estimate the borders of the OA damaged area, providing other important information useful in formulating a more precise diagnosis.

The results obtained in the classification of the OA grades were used to build up a supervised machine learning algorithm based on LDA to automatically discriminate between resections in healthy conditions and those affected by the four different abovementioned pathological phenotypes. The global accuracy of the algorithm in the single-point measurement was found to be about 86%, but it reaches higher values for the minimal (92%), moderate (96%) and severe (100%) grades. It worth noting that without the PCA reduction of dimensionality we report a slightly lower value of 84% in the global accuracy. However, even if the difference in the performance is low, the use of PCA is advantageous as data-mining technique to help the interpretation of the most important features for classification and, as a reductional of dimensionality, to reduce the calculation time for the model, especially when the size of the dataset increases. The future possibility to couple the in vivo characterization using Brillouin spectra through endoscopic probes^40^, also using measurements in the time range of a few seconds, with an efficient machine learning method may pave the way for the technique to be used intraoperatively even by personnel not trained in the use of spectroscopic techniques.

However, the results obtained in this proof-of-principle will have to be further validated by a pre-clinical study considering (i) a more extended number of OA samples analyzed to develop an appropriate database of spectra, (ii) to extend the analyses on healthy samples collected from cadaver donors without signs of OA, increasing the inter-individual variability to have a comprehensive grading of the pathology, and (iii) to employ fresh/frozen sections to reproduce an environment more similar to the in-vivo condition. Concerning this, recent results obtained by our group^28^ revealed that the paraformaldehyde (PFA) fixation, followed by the storage ethanol, can modify the local composition of the tissue due to the loss in its lipidic content so that the use of frozen samples likely will increase the efficacy of the algorithm, due to higher accessibility to the characterization cellular component. In the present study, it is worth noting that the same samples were first subjected to BRmS and then analysed by histological protocols. Therefore, since BRmS technique requires multiple analyses repeated over time, the fixation protocol was the only way to avoid the sample contamination due to aerobic bacteria and fungi present in the indoor air environment, which could seriously alter the architecture and biochemical properties of bone/cartilage tissues and cause misleading histological results.

Last but not least, we stress that this proof-of-concept study provides a suitable approach, that can be potentially extended to other degenerative pathologies of the musculoskeletal system, as well as to cancer and bone infections diagnosis, upon the identification of appropriate mechanical and/or chemical markers and the gathering of a spectral dataset, suitable for the development of artificial intelligence models.

## Materials and methods

### Patients and sample collection

This study complies with ethical rules for human experimentation stated in the 1975 Declaration of Helsinki. The approval by the Ethics Committee of the Area Vasta Emilia Centro—Regione Emilia-Romagna (CE-AVEC) was obtained (43/2019/Sper/IOR; EM103-2021_43/2019/Sper/IOR_EM1). Written informed consent was obtained from all individual participants included in the study. Patients referred for this study underwent unilateral total hip arthroplasty (UTHA) and were screened for eligibility according to the principle of inclusion and exclusion criteria, as reported in Table 1. The American Society of Anesthesiologists (ASA) physical health status classification^41^ is used as inclusion/exclusion criteria. Specifically, patients with some comorbidities, such as diabetes, chronic renal failure, and heart and respiratory diseases are excluded (i.e., ASA ≥ 3).

**Table 1:** Principle of inclusion and exclusion criteria.

Inclusion criteria	Exclusion criteria
Male or female between the age of 45 and 70 years	Lack of written consent
ASA score ≤ 2	ASA score ≥ 3
Severe osteoarthritis in the supero-lateral region (K-L 3–4) of the femoral head with a healthy or less pathological infero-medial region (K-L 0–2)	Patients with cognitive impairments and psychiatric diseases

**Figure 7:**
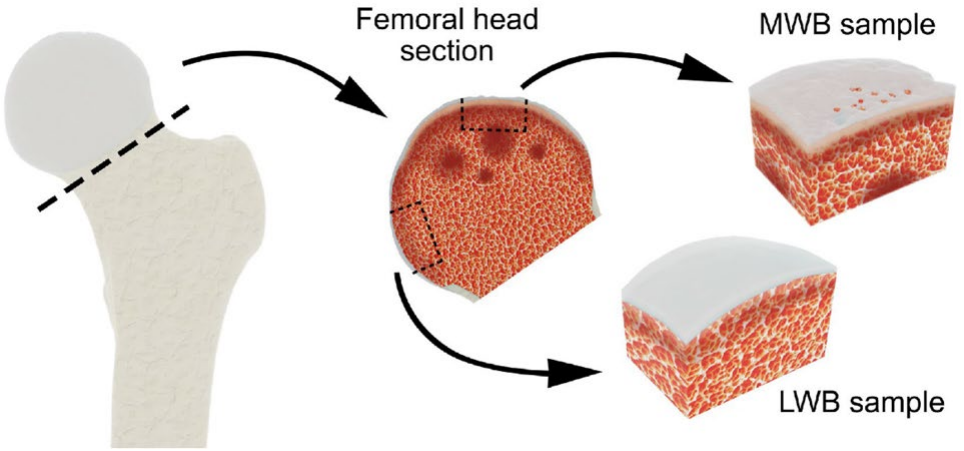
Graphical sketch depicting the femoral head samples collected from patients enrolled in the study. A femoral head was excised from each patient who underwent unilateral total hip arthroplasty. A most weight-bearing sample (MWB) was procured from the supero-lateral region. A least weight-bearing sample (LWB) was procured from the infero-medial region.

A total of 10 samples (two per patient) were procured from 5 patients (denoted in the text as biopsy#1 to biopsy#5), four men and one woman with a mean age of 55.6 years (45–67). Specifically, two sections, ranging between 1 to 2 cm in thickness and width, were obtained from each patients’ femoral head as shown in Fig. 7 and collected as waste surgical material. One sample was procured from the infero-medial region (referred to as the least weight-bearing surface, LWB) and one from the supero-lateral region (referred as to the most weight-bearing surface, MWB). Then, each sample was fixed in a solution of 4% paraformaldehyde (PFA; Sigma-Aldrich, Milan, Italy) for 24 h, washed in distilled water and stored at room temperature in a solution of 70% ethanol. Before measurement, the sample was extracted from ethanol and air-dried.

### Histological and histomorphometric analyses

Samples were processed for paraffin embedding after the spectroscopic analysis. Specifically, samples were decalcified in a 5% solution of Formic (ACEF, Fiumicino, Rome, Italy) and 4% Hydrochloric acid (Sigma-Aldrich, Saint Louis, Missouri, USA) for an average of 15 days, dehydrated in increasing ethanol solutions, defatted in xylene (VWR International, Milan, Italy) and finally paraffin-embedded (Sigma-Aldrich, Saint Louis, Missouri, USA). 5-µm thick sections were cut by a semi-automated microtome (H340E, Microm International GmbH, Walldorf, Germany) and stained with Safranin-O (Safranin O, Polysciences, Inc., Warrington, Pennsylvania USA) and Fast Green (Fast Green FCF, Histo-Line Laboratories, Milan, Italy). Histological images of the samples’ sections were taken with a digital pathology slide scanner (Aperio Scanscope AT2, Leica Biosystems, Wetzlar, Germany)^42^. The slides stained were scored with the histologic-histochemical grading system proposed by Mankin et al. which assesses four parameters, cartilage structure, cellularity, Safranin-O staining and tidemark integrity. Each parameter has subcategories, and the scores are summed to provide a total score ranging from 0 (normal) to 14 (most severe OA). Specifically, the sections were analysed for abnormalities in the structure such as surface irregularities or pannus presence (score 1 and 2), clefts from transitional to calcified zone (score 3–5) and complete disorganization (score 6); cell population (from normal—score 0 to hypocellularity—score 3); Safranin-O stain distribution (scores 0—4) and tidemark integrity (intact—score 0 or crossed by blood vessels—score 1)^43^.

### Brillouin and Raman micro-spectroscopy instrumental set-up

The Brillouin and Raman micro-spectroscopic setup consists of a 532 nm single-mode solid-state laser, a polarizing beamsplitter (PB) which reflects the laser light into a microscope objective lens for imaging and mapping. The sample is mounted on an xyz translation stage. The inelastically scattered light is collected using the same objective in a backscattering geometry and then split in frequency and direction by an edge filter (EF1), so that the Stokes component (> 30 cm^−1^) is sent to an iHR320 Triax Raman monochromator (Horiba, Kyoto, Japan) and the quasi-elastic and anti-Stokes components are sent to a high contrast multipass tandem Fabry–Perot interferometer (TFP-2 HC, JRS Scientific Instruments, Zürich, Switzerland). More details of the instrumentation can be found in Ref.^17^. The microscope objective selected for the measurements of the femoral head resections is a 20 × objective (NA 0.42) so that, a volume of about 2 μm for about 10 μm in depth is enlightened by the laser beam. The measurements were acquired in regions of interest (ROIs) in both the articular cartilage surface (xy plane of Fig. 1A) and the subchondral bone below (xz plane of Fig. 1B) of the five couple of resections described before in patients and sample collection. Laser power was filtered to about 7 mW to avoid tissue photodamage and the acquisition time was set to 50 s for each spectrum acquired on biopsy#1 to biospy#4 and to 10 s for spectra acquired on biopsy#5. Maps performed on biospy#2 were acquired considering a 3 μm step in the x, y and z axes.

### Brillouin data analysis

Brillouin Light Scattering arises from the interaction of the incident light with acoustic waves, spontaneously propagating in the matter due to molecular translations. Brillouin peak’s intensity is proportional to the concentration of the elastic species supporting the acoustic mode, while the square of its frequency shift ν_B_ is related to the longitudinal elastic modulus M, and thus to tissue stiffness, through the relation:

1 $$M=\frac{{\upsilon }_{B }^{2} {\lambda }^{2}\rho }{4{n}^{2}},$$

where ρ is the mass density and n is the refractive index of the medium, and λ is the wavelength of the laser source^18,44^. Figure 1C reports typical Brillouin spectra (before the break) collected in the articular cartilage surface (NCC-blue), the cortical (SB-red) and the trabecular (TB-black) portions of the subchondral bone, revealing the existence of almost two well-separated peaks in the same scattering volume, namely P_SOFT_ (between 4 and 13 GHz) and P_HARD_ (between 13 and 34 GHz). This peculiar behaviour, ascribable to the coexistence of sub-micrometric aggregates with a different mechanical phenotype, has been widely documented by our group in several samples of musculoskeletal tissues^26–29^. Brillouin maps in Figs. 2 and 3, reporting the relative percentage of P_SOFT_ and P_HARD_ components along with their frequency shift, were obtained by computing peaks’ spectral moment analysis following the procedure reported in Ref.^27^ since this approach to data analysis has proven to be particularly well suited for obtaining average information on tissues that are very heterogeneous in the Brillouin spectral shapes^45^. After that, the intensity of the peaks was normalized to obtain the relative intensities I_SOFT_ and I_HARD_, considering both the different scattering efficiency of the two peaks and a filling parameter that estimate the quantity of the scattering volume effectively occupied by the sample, following the procedure in Refs.^27,29^. This procedure is strongly necessary for maps comparisons since the intensity of each peak is dependent upon the quantity of material enlightened by the laser beam and both the cartilage and the bone resections are characterized by a rough surface. Finally, the average frequency shift of each peak (${\overline{v} }_{B}$) were calculated using:

2 $${\overline{v} }_{B}={\sum }_{i}{I}_{i}{\upsilon }_{i} /{\sum }_{i}{I}_{i},$$

where ${I}_{i}$ is the intensity and the index $i$ spans spectral channels in the range 4–13 GHz (low-frequency, ν_SOFT_) and 13–34 GHz (high-frequency, ν_HARD_)^45^.

### Principal component analysis and machine learning modelling based on linear discriminant analysis

Principal component analysis (PCA) was applied to the Brillouin spectra collected in different regions of interest to reveal trends in the spectral features useful for the classification of the distinct mechanical phenotypes. PCA is an unsupervised method used to reduce the dimensionality of the dataset, by identifying combinations of variables (the Brillouin intensities at each frequency) more involved in the description of the spectral variance^38,39^. The information obtained from the PCA analysis was used to build up a machine learning (ML) algorithm based on Linear Discriminant Analysis (LDA). LDA is a generalization of Fisher's linear discriminant, finding a linear combination of variables that characterizes or separates known classes of objects (supervised method)^39^. PCA and the LDA-based machine learning algorithm were performed in the Rstudio environment (www.rstudio.com) using the basic R package for the PCA algorithm and “mass”,“caret”, “e1071”, and “ROCR” R packages for the ML-LDA model, respectively. Data pre-processing was performed using the basic and the “Tidyverse” R packages. In Fig. 5, the scores were plotted using the R package Plotly.

### Raman data analysis

Raman Light Scattering deals with the interaction of light with the vibrational modes of the macromolecules present in the sample, thus unveiling its chemical composition. Raman peaks’ intensity depends on the concentration of the chemical species, whilst their frequency shift is due to the nature of the oscillators present in the scattering volume^33,46^. Typical Raman spectra collected in the human femoral resections are reported in Fig. 1C (after the break) after a baseline removal correction via a polynomial fit to reduce the fluorescence background. Important peaks of interest are assigned in Fig. 1C to facilitate the comparison between different tissues. In particular, the hydroxyapatite vibration of the phosphate group (ν_1_PO_4_^3−^) at 965 cm^−1^ is generally used to assess (i) the bone mineralization degree when compared to a more general signal from the organic component, such as the CH_2_ wagging at 1445 cm^−1^, and (ii) the carbonate-to-phosphate ratio when compared to carbonate substitution vibration at 1064 cm^−1^. Characteristic signatures of proteins are the amide III and amide I bands, relative to vibrations specific to the peptidic bond at 1245 and 1660 cm^−1^, whereas typical signals from the lipids can be found at 1301 cm^−1^ and 1780 cm^−1^ due to fatty acids and esters respectively. In addition to that, also the multiple-peaked CH_2_–CH_3_ band at 2800–3100 cm^−1^ can be used to evaluate the lipids-to-proteins ratio since the CH_2_ stretching of lipids is centred at about 2872 cm^−1^, whereas the CH_3_ stretching of proteins occurs at 2945 cm^−1^^34–36^.

## Supplementary Information

Supplementary Information.

## Data Availability

All data needed to evaluate the conclusions in the paper are present in the paper and/or the Supplementary Materials. Additional data related to this paper may be requested from the corresponding author.
