# Supplementary material for: Brillouin–Raman micro-spectroscopy and machine learning techniques to classify osteoarthritic lesions in the human articular cartilage
Source: Sci Rep. 2023 Jan 30;13:1690. doi: 10.1038/s41598-023-28735-5 (PMC9886972; doi:10.1038/s41598-023-28735-5)
Supplement: Supplementary file 1 — Supplementary Information. [file 41598_2023_28735_MOESM1_ESM.pdf]

Brillouin-Raman micro-Spectroscopy and Machine Learning techniques to classify osteoarthritic lesions in the human articular cartilage.

Macroscopic evaluation of LWB and MWB of biopsy#2.

Fig. S1 reports the photographs of the LWB and the MWB samples excised from biopsy#2 used for the macroscopic evaluation of OA degeneration, considering the view on the articular surface (plane xy – Fig. S1A) and the subchondral bone below (plane xz – Fig. S1A). The morphological differences between the LWB (Fig. S1B) and the MWB (Fig. S1C) appear evident in both cases since weight-bearing is non-uniform and spatially specific. Therefore, compared to LWB, MWB being subjected to long-term cyclic stress it is featured by the reduction of cartilage surface magnitude as well as the development of degenerative loci in the subchondral bone.

Comparison of LWB and MWB sections of additional biopsies.

Fig. S2 reports the PCA analysis of Brillouin spectra collected on the articular cartilage surface of LWB and MWB samples of biopsy#3 (Fig S2A), #4 (Fig S2B) and #1 (Fig S2C) along with their histological assessment on the xz plane through Safranin-O/Fast Green staining (respectively Fig S2D, Fig. S2E and Fig. S2F). PCA score plots reveal that in both the #3 and the #4 cases Brillouin spectra analysis originates distinct clusters, thus demonstrating that the mechanical investigation is sufficient to discriminate the properties of the areas subjected to major (MWB-red points) and minor loadings (LWB-black points). Furthermore, the correspondent loadings plots summarize the main differences in the spectrum of the two zones, disclosing that in both cases the MWB areas (red) are characterized by a small shift to lower frequency values (yellow stars) of the not-mineralized collagen with respect to the LWB areas (black). This fact can be tentatively ascribed to a more-disordered organization in the not-mineralized collagen, due to a propagation of the disease into the cartilage layer. The histological analysis in Fig. S2C and S2D for LWB and MWB areas of biopsy#3 (Mankin: LWB: 7; MWB: 7,5) and #4 (Mankin: LWB: 2,5; MWB: 8,5) respectively, confirms that the region most subjected to loadings are also the ones presenting a more advanced grade of the pathology. In comparison to LWB samples, clefts to radial and calcified zone, clusters formation, hypocellularity and a slight reduction in Safranin-O staining were observed. Furthermore, tidemark integrity resulted compromised due to doubling phenomenon or interruptions because of blood vessels. Conversely, the PCA score plot of biopsy#1 (Fig. S2C) shows that the clusters of LWB (black-spectra) and MWB (red-spectra) are almost overlapped, revealing similar mechanical characteristics in the portion. However, a small shift towards negative values of the MWB points PCA score plots with respect to the second principal component can be appreciated. The loading plot on the right suggests that this can be attributed to the presence of a higher contribution from the P_{SOFT} component (i.e., the cellular content and the not-ordered phase of ECM), suggesting an initial phase of remodelling. This is confirmed by the histological assessment in Fig. S2F (Mankin: LWB: 5; MWB: 12). It is worth noting that in these three cases no sign of ossification of the articular surface is detectable in the Brillouin spectrum, thus indicating an overall pathologic progression less advanced than in the MWB section of the previously analyzed biopsy#2.

Staging evaluation on the articular cartilage and the subchondral bone of a single patient biopsy.

The presence of different micrometric areas with distinct phenotypes stresses another important concept to be taken into consideration in the pathology development–i.e., the OA staging. In

fact, the diagnosis of the pathologic status of the articular cartilage involves the evaluation of both the severity (grade) and the extension of the lesion (stage), since the pathology could present a certain spectrum of cases, whose extremes are extremely localized but very severe lesion (high grade and low stage) and an extremely extensive but mild lesion (low grade and high stage) [1]. Therefore, the analysis of different portions of the supero-lateral region of the articular cartilage surface during the arthroscopic procedure is an essential element both to evaluate the average degree of severity of the OA insult and to assess its overall extent.

Fig. S3 reports the PCA analysis of Brillouin spectra (BLS) collected on different ROIs (namely A - red, B - black, C - blue, D - green, E - orange and S - brown, Fig S3A) in the articular cartilage surface of MWB sample (biopsy#2), along with the spectra collected on the articular surface of the section selected as healthy phenotype (LWB of biopsy#1, Healthy - magenta). Specifically, the score plot shown in Fig. S3B demonstrates that the BLS collected on the top of the section in Fig. S3A)–i.e. A, B, C, D and S regions of interest (ROIs)–share common features with respect to PC2. Conversely, the ROI in the bottom of the section, evidenced by an orange circle (E) in the photograph, has a particular spectral shape more similar to the healthy phenotype (healthy). The graph of the averaged spectra of each ROIs (Fig. S3C) helps to understand better the origin of this clustering: the ROIs in the top are characterized by BLS with a clear signal coming from the mineralized collagen bundles (ν_{HARD} has values between 19 up to 24 GHz), revealing a severe grade of OA progression with the exposition of the subchondral bone. Going from the top of the sample down to the E region (Fig. S3A) the OA grade becomes less advanced and the average spectrum presents only signals from the non-mineralized collagen bundles.

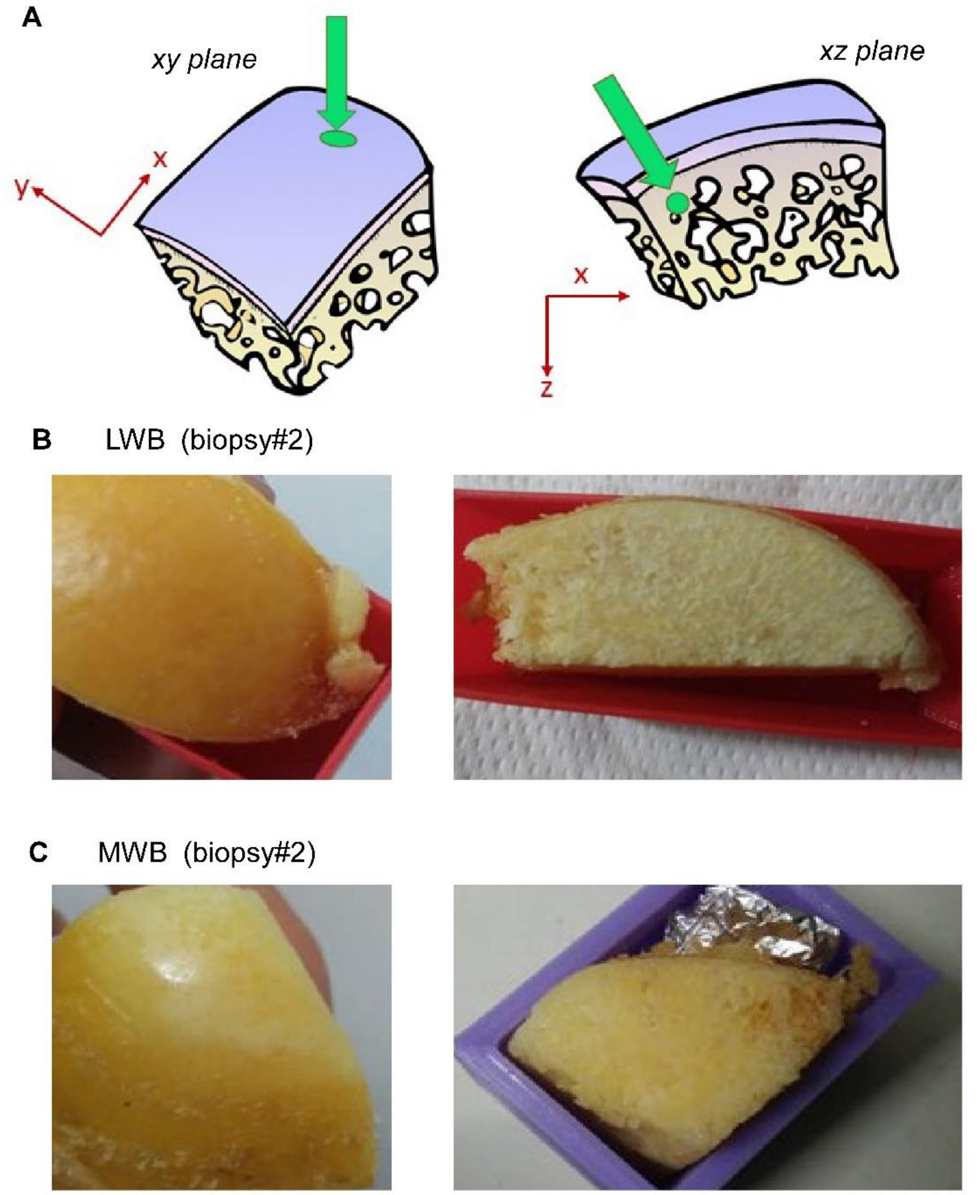

Figure S1. (A) Graphical sketches depicting the articular cartilage surface (i.e., the xy plane) and the subchondral bone below (i.e., the xz plane). Views of the (B) LWB and C) MWB samples of biopsy#2 used for the macroscopic evaluation of OA degeneration.

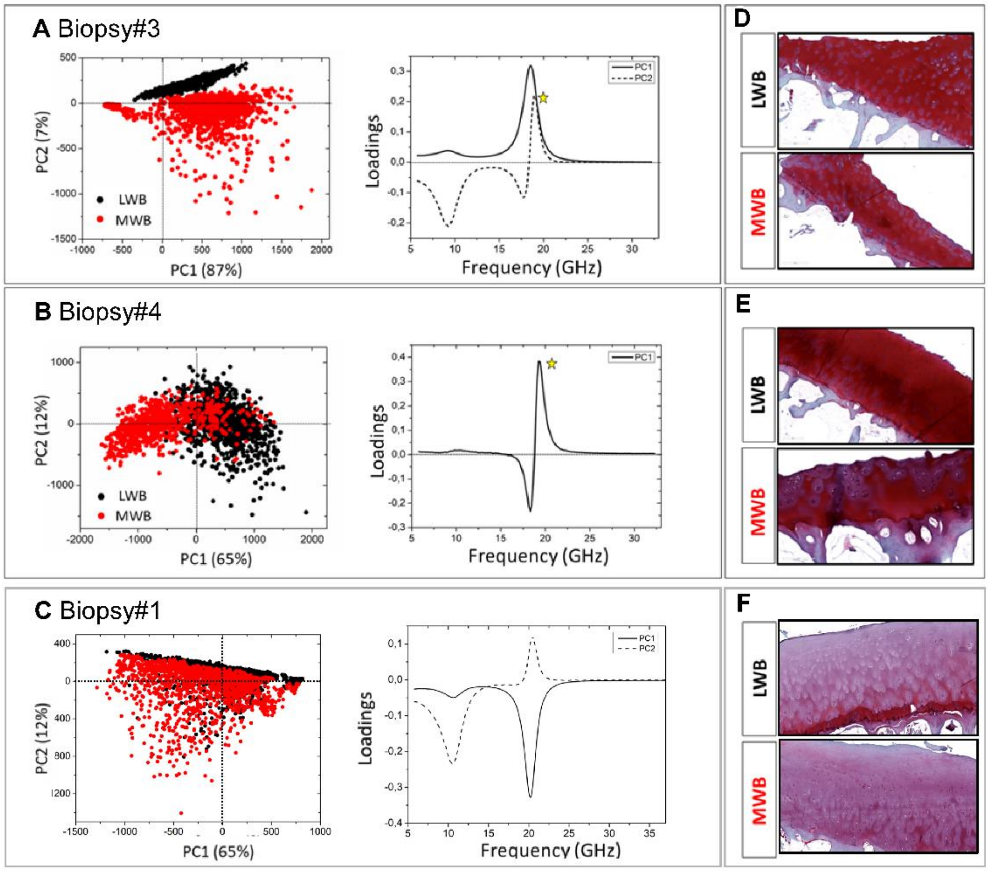

Figure S2. Comparison between BLS collected in the least weight-bearing (LWB-black) and most weight-bearing sample (MWB-red) in the articular surfaces of different biopsies (biopsy#3, biopsy#4 and biopsy#1). PCA scores and loadings plot of BLS collected in the least weight-bearing (LWB-black) and most weight-bearing (MWB-red) areas of (A) biopsy#3, (B) #4 and (C) #1 articular surfaces, along with their respective histological assessments (D, E and F) through Safranin-O/Fast Green.

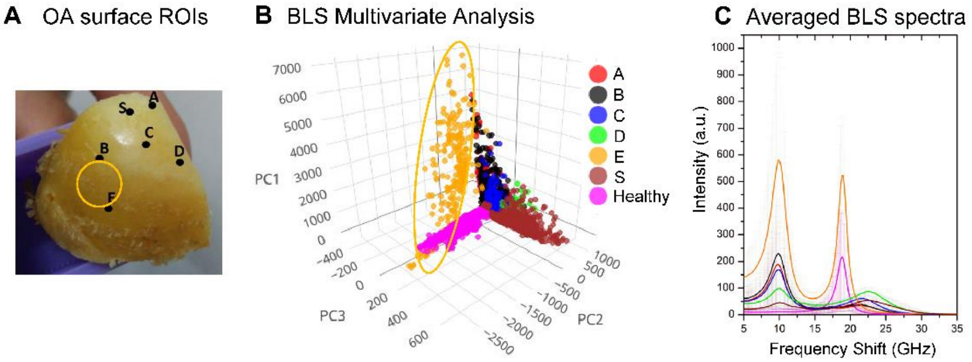

Figure S3. (A) Photograph of the articular cartilage surface of the MWB sample (biopsy#2) reporting some ROIs where the BLS were collected. (B) PCA score plot of Brillouin spectra detected in the different ROIs (A - red, B - black, C - blue, D - green, E - orange, S – brown) along with the one collected on the healthy phenotype–i.e., the LWB of biopsy#1 (healthy – magenta). (C) Averaged BLS spectra of each ROI.

Reference

1. K. P. Pritzker, S. Gay, S. A. Jimenez, K. Ostergaard, J. P. Pelletier, P. A. Revell, D. Salter, and W. B. van den Berg, "Osteoarthritis cartilage histopathology: grading and staging," Osteoarthritis Cartilage **14**, 13-29 (2006).
